# Comparing the clinical practice and prescribing safety of locum and permanent doctors: observational study of primary care consultations in England

**DOI:** 10.1186/s12916-024-03332-z

**Published:** 2024-03-27

**Authors:** Christos Grigoroglou, Kieran Walshe, Evangelos Kontopantelis, Jane Ferguson, Gemma Stringer, Darren M. Ashcroft, Thomas Allen

**Affiliations:** 1 Manchester Centre for Health Economics, Division of Population Health, Health Services Research and Primary Care, University of Manchester, Manchester, UK; 2 Alliance Manchester Business School, University of Manchester, Manchester, UK; 3 NIHR School for Primary Care Research, Centre for Primary Care, Division of Population Health, Health Services Research and Primary Care, University of Manchester, Manchester, UK; 4 Division of Informatics, Imaging and Data Sciences, University of Manchester, Manchester, UK; 5 Health Services Management Centre, University of Birmingham, Birmingham, UK; 6 NIHR Greater Manchester Patient Safety Research Collaboration, Division of Pharmacy and Optometry, University of Manchester, Manchester, UK; 7 Centre for Pharmacoepidemiology and Drug Safety, School of Health Sciences, School of Health Sciences, Faculty of Biology, Medicine and Health, University of Manchester, Manchester, UK; 8 Danish Centre for Health Economics, University of Southern Denmark, Odense, Denmark

**Keywords:** Locum doctors, Quality, Patient safety, Electronic health records, Medical workforce, General practice

## Abstract

**Background:**

Temporary doctors, known as locums, are a key component of the medical workforce in the NHS but evidence on differences in quality and safety between locum and permanent doctors is limited. We aimed to examine differences in the clinical practice, and prescribing safety for locum and permanent doctors working in primary care in England.

**Methods:**

We accessed electronic health care records (EHRs) for 3.5 million patients from the CPRD GOLD database with linkage to Hospital Episode Statistics from 1st April 2010 to 31st March 2022. We used multi-level mixed effects logistic regression to compare consultations with locum and permanent GPs for several patient outcomes including general practice revisits; prescribing of antibiotics; strong opioids; hypnotics; A&E visits; emergency hospital admissions; admissions for ambulatory care sensitive conditions; test ordering; referrals; and prescribing safety indicators while controlling for patient and practice characteristics.

**Results:**

Consultations with locum GPs were 22% more likely to involve a prescription for an antibiotic (OR = 1.22 (1.21 to 1.22)), 8% more likely to involve a prescription for a strong opioid (OR = 1.08 (1.06 to 1.09)), 4% more likely to be followed by an A&E visit on the same day (OR = 1.04 (1.01 to 1.08)) and 5% more likely to be followed by an A&E visit within 1 to 7 days (OR = 1.05 (1.02 to 1.08)). Consultations with a locum were 12% less likely to lead to a practice revisit within 7 days (OR = 0.88 (0.87 to 0.88)), 4% less likely to involve a prescription for a hypnotic (OR = 0.96 (0.94 to 0.98)), 15% less likely to involve a referral (OR = 0.85 (0.84 to 0.86)) and 19% less likely to involve a test (OR = 0.81 (0.80 to 0.82)). We found no evidence that emergency admissions, ACSC admissions and eight out of the eleven prescribing safety indicators were different if patients were seen by a locum or a permanent GP.

**Conclusions:**

Despite existing concerns, the clinical practice and performance of locum GPs did not appear to be systematically different from that of permanent GPs. The practice and performance of both locum and permanent GPs is likely shaped by the organisational setting and systems within which they work.

**Supplementary Information:**

The online version contains supplementary material available at 10.1186/s12916-024-03332-z.

## Background

Staff shortages in the global health sector have been described as one of the most significant health issues of our time [1]. Recruitment difficulties, high vacancy rates, and low retention [2] have combined to result in increasing reliance on temporary staff [3]. Despite international growth in the number of temporary doctors (often known as locums or locum tenens) [4–6] there has been limited research on how the use of locum doctors might affect patient safety and quality of care [7].

Locums are a vital resource that enables healthcare organisations to deliver care; however, the way locums are recruited, employed and used by organisations may to have implications for quality and safety [8]. There have been some past high-profile examples of poor-quality care by locum doctors [9–11], the same could likely be said for permanent doctors, but comparative research between the two groups is lacking. However, previous research has shown that locums are often stigmatised, blamed for quality problems and treated with suspicion and even hostility by permanent doctors and other clinical staff [12]. The use of locum doctors in healthcare has also been associated with lower productivity [13] and higher costs [14]. But previous research is extremely limited and robust evidence about the quality and safety of locum doctors’ practice is lacking partly due to the poor availability of routinely collected data about locum doctors [8].

The aim of this research was to examine whether there exist differences in clinical practice and prescribing safety outcomes for locum and permanent doctors working in primary care in England using a unique database that allows for the distinction between locum and permanent general practitioners (GPs). This paper addresses a gap in the empirical evidence base by seeking to compare a range of quality and safety outcomes for locum and permanent general practitioners. These included practice revisits; prescribing, test ordering and referral rates; and subsequent accident and emergency attendances and emergency hospital admissions. It also compares permanent and locum doctors on a number of established indicators of prescribing safety.

## Methods

### Data sources and study population

We used the Clinical Practice Research Datalink (CPRD) GOLD, a large computerised database of anonymised primary care medical records. It contains complete electronic health records (EHRs) for over 14 million patients in general practices using the Vision system, with the healthcare events (diagnoses, treatments, referrals, tests and prescriptions) recorded using coding systems [15]. The database is broadly representative of the United Kingdom’s (UK) population in terms of age, gender and deprivation, and the data have been shown in numerous validation studies to be generally of high quality [16, 17]. However, larger practices are slightly over-represented and the data are from practices using the Vision clinical system, and clinical system usage is geographically clustered in the UK [18]. Practices need to meet pre-specified data entry quality criteria to be defined as ‘up to research standard’, and for each study year, our main sample included all CPRD English practices that were classed as such for the whole year. We used all eligible patients in CPRD GOLD for the years 2010–2011 to 2021–2022.

We also obtained CPRD-linked Hospital Episode Statistics (HES) data. The national Hospital Episode Statistics (HES) data contain details of all admissions and A&E visits to NHS hospitals in England [19]. Area deprivation, as measured by the Index of Multiple Deprivation (IMD) 2015 was available at the 2011 Lower Super Output Area (LSOA) level, a level of English geography with approximately 1,500 residents. The IMD measures deprivation at the area level based on domains, such as income, employment, health, housing and general environment and is the most complete and widely used approach to quantify relative deprivation and affluence for small areas in England [20]. From CPRD, we obtained patient-level quintiles of deprivation.

Linkage between CPRD GOLD data and the IMD and HES data sources is available at the individual patient level for those patients registered at practices in England that have consented to data linkage. Linkage between data sets is undertaken by CPRD using a deterministic linkage algorithm, based on a patient's exact NHS identification number, sex, date of birth, and residential postcode and approximately 68.6% of patients were eligible for linkage with the majority of the remainder 31.4% living in the other constituent UK countries [21].

### Outcomes

To compare the clinical practice and prescribing safety of locum and permanent doctors we used a range of outcome measures based on face-to-face consultation events with either a locum or permanent GP (consultations with other staff groups were not included). The clinical codes used to generate these outcomes are included in Additional file 1 (Tables S1–S15). These outcomes are widely used to compare clinical practice and prescribing safety and were selected on the basis of their relevance to patient quality and safety and the work locums undertake.

Return to practice for a revisit within 7 days, was selected as a general quality and safety measure under the assumption that a patient who revisits their practice within a week may not have been assured or satisfied by the initial consultation. Hospital visit outcomes are often used as performance indicators and good quality, accessible and continuous primary care may prevent the development of health problems that require an A&E visit or an emergency hospital admission, particularly admissions for ambulatory care sensitive conditions (ACSCs) [8, 22–24]. Referral and test ordering rates were included to assess whether differences existed, though the interpretation of any such differences is complex [25, 26]. Prescribing rates (including repeat prescriptions) for some drug groups were measured as, in the UK, national guidelines have stressed the need to control and reduce the use of antibiotics, [27] strong opioids, [28] and hypnotics [28]. Some well-established measures of prescribing safety were included based on validated indicators aimed at reducing rates of hazardous prescribing [29].

#### Practice revisit within 7 days

Our first outcome examined whether the patient revisited the general practice within 7 days of a consultation event. We identified consultation events within CPRD for each patient in each year, and we calculated the time in days between two consecutive consultation events including telephone and online consultations. We generated a binary variable indicating whether the patient revisited for a consultation within 7 days.

#### Antibiotic prescriptions

Antibiotic prescriptions were classified using the British National Formulary (BNF) sections (Additional file 1: Table S1). For all consultation events, we created a binary variable indicating whether an antibiotic was prescribed during the consultation.

#### Strong opioid prescriptions

Strong opioid prescriptions (alfentanil, buprenorphine, cyclizine, diamorphine, methadone, morphine, naloxone, oxycodone, papaveretum, pentazocine, pethidine, tapentadol) were classified using the BNF sections (Additional file 1: Table S2). For all consultation events, we created a binary variable indicating whether a strong opioid was prescribed during the consultation.

#### Hypnotics and anxiolytics prescriptions

Prescriptions for hypnotics were classified using the BNF sections (Additional file 1: Table S3–S4). Benzodiazepines and z-drugs (zolpidem and zopiclone) were included in the analyses. For all consultation events, we created a binary variable indicating the prescription of a hypnotic during the consultation.

#### A&E visits

Using the HES A&E data, we identified all A&E visits within 7 days following a consultation event. Two binary variables were created indicating whether there was an A&E visit on the same day or within 1 to 7 days of the consultation event.

#### Emergency admissions

Emergency admissions are recorded in the HES Admitted Patient Care. We identified all emergency admissions within 7 days following a consultation event. Two binary variables were created indicating whether there was an emergency admission on the same day or within 1 to 7 days of the consultation event.

#### Ambulatory Care Sensitive Conditions (ACSC) admissions

Classification of ACSC hospital admissions for the study used the International Classification of Diseases, 10th edition (ICD-10) and included all hospital admissions with a primary diagnosis related to one of the nine ACSCs that are incentivised in the UK’s Quality and Outcomes Framework (QOF) [30]. We identified all ACSC admissions within 7 days following a consultation event. Two binary variables were created indicating whether there was an ACSC admission on the same day or within 1 to 7 days of the consultation event. The ICD-10 chapters used to define admissions for ambulatory care-sensitive conditions are provided in Table S16 in Additional file 2.

#### Tests

We identified all consultation events and created a binary variable indicating whether any test was ordered during the consultation event.

#### Referrals

We identified all consultation events and created a binary variable indicating whether a referral to any other service was made during the consultation event.

#### Prescribing safety indicators

We adapted 10 indicators of prescribing safety developed for PINCER, a pharmacist-led intervention to improve prescribing safety by identifying patients at risk of potentially hazardous prescribing events [31, 32]. These indicators are associated with potentially harmful outcomes such as GI bleeding, asthma, heart failure and stroke. The code lists used to define product and medical codes for the potentially hazardous prescribing indicators are provided in Additional file 1: Tables S5–S15.

### Statistical analyses

We conducted an observational study of GP consultations of registered patients at 407 CPRD GOLD participating general practices in England, between 1st April 2010 to 31st March 2022.

Consultation information was extracted within each financial year, for each active patient (registered for at least 1 day during the respective year). Patients who had a recorded year of death before the beginning of the period of study were excluded from the analyses. Patients who had a consultation following their date of death as recorded within CPRD were excluded from the analyses. We restricted our sample to include only practices in England, as data on the IMD and hospital outcomes were only available for patients located in England.

#### Clinical practice

In the first set of models investigating various clinical practice indicators, we randomly selected one consultation event for each patient within each financial year, aligning all the patient outcomes and covariates to that specific event date. This allowed us to give equal weights to patients and limited the potential for confounding introduced by higher-need patients who may be visiting numerous times within a year. Our exposure was a binary variable indicating whether the consultation was by a permanent GP or a locum GP. We were able to identify permanent GPs and locum GP through the staff role field which is available for every consultation. This approach was used for practice revisits; prescribing of antibiotics, strong opioids and hypnotics; tests and referrals; and hospital outcomes.

#### Prescribing safety indicators

In the second set of models investigating the PINCER prescribing safety indicators, for each indicator, we identified all consultation events with patients who could be exposed to potentially hazardous prescribing, because of a specific diagnosis or prescription on the day of the consultation (i.e. index event). These events were split into consultations by locum or permanent GPs. Second, for each index consultation event, we looked at consultation events during a pre-specified time window (which varied across indicators; Table S17 in Additional file 2) leading up to the index event, to identify pre-existing prescriptions or conditions that would trigger a potentially hazardous prescribing outcome when combined with the index event. For each index consultation event, a binary variable indicated whether potentially hazardous prescribing was triggered. This allowed us to operationalise rates of potentially hazardous prescribing events for both locum and permanent GPs. Our exposure was again a binary variable indicating whether the consultation involved a permanent GP or a locum GP and we aligned patient covariates to the index consultation event using unique patient IDs.

For example, for indicator A we identified consultations for patients who were over 65, at which they were prescribed an NSAID. We then identified those patients who were not also prescribed the recommended proton-pump inhibitor (PPI) or H2 receptor antagonist at the consultation or in the preceding 3 months. The operational definitions for the PINCER prescribing safety indicators are provided in Table S17 in Additional file 2.

#### Model covariates

We used Read codes [33] to identify the presence of comorbidities and we calculated the validated Cambridge Multimorbidity Score [34] for each patient in our cohort in 2010, which was our baseline year. Additional information on patient age, gender, years registered with the practice, practice list size, patient urban/rural location, patient deprivation and region was used.

We employed multi-level mixed effects logistic regression models to quantify the association between the exposure of interest (locum/permanent GP) and the outcomes of interest, controlling for all available covariates over time. For the first set of models for clinical practice outcomes, our analyses used a random consultation per patient and accounted for the nested structure of the data: patients within general practices, within regions. For the prescribing safety indicators models, analyses were conducted on repeated consultations, with consultations nested within patients. We included random effects for practices and fixed effects for regions in both sets of models. In both sets of models, consultation events with missing information on age or gender were excluded from the analyses. We also performed a sensitivity analyses excluding the last 3 years of data, to evaluate whether the effect sizes of our exposure (ie locum consultations) on the outcomes were affected by the COVID-19 period in the UK.

Stata v17 was used for data cleaning, management and analyses and an *α* level of 1% was used throughout [35]. However, statistical significance is not very informative in analyses of datasets of this size and we focus on the clinical significance of the effect sizes rather than *p* values [35].

## Results

The number of practices in England participating in CPRD GOLD varied from 487 in 2010–2011, to 228 in 2015–2016, to 42 in 2021–2022. Of these, only 42 practices contributed data throughout the whole of the study period and 407 had complete data, including hospital admissions and deprivation for their patients. For the first set of models, our cohort consisted of 3,591,367 patients with 13,696,455 recorded consultations between 407 practices across all years. For the second set of models, our cohort consisted of 547,146 patients with 7,623,205 recorded consultations, which varied by indicator and included patients from 407 practices across all years. In Table 1, we provide descriptive statistics for the consultation outcomes and some important practice and patient characteristics. In Table 2, we summarise the numerators and denominators that allowed us to calculate the proportion of consultations that were exposed to potentially hazardous prescribing for each indicator. The numerator is the number of consultations that were exposed to each type of potentially hazardous prescribing, and the denominator, is the number of consultations of patients at risk of exposure to the hazardous prescribing indicator.

**Table 1:** Descriptive statistics on patient outcomes and patient and practice characteristics, over 2010–2011 to 2021–2022

	**Permanent GPs**	**Locums**
**Number of patients at risk (percent)** **(*N* = 13,696,455 consultation events)**		
Practice revisits within 7 days	1,008,934 (8.18%)	94,204 (6.89%)
Antibiotic prescriptions	1,114,621 (9.04%)	144,724 (10.58%)
Strong opioid prescriptions	278,819 (2.26%)	31,535 (2.31%)
Hypnotics prescription	105,928 (0.86%)	10,488 (0.77%)
Same day emergency admissions	20,048 (0.16%)	2,120 (0.16%)
Same day A&E visits	37,861 (0.31%)	4,723 (0.35%)
Same day ACSC admissions	19,084 (0.15%)	2,030 (0.15%)
Emergency admissions within 1–7 days	18,217 (0.15%)	1,929 (0.14%)
A&E visits within 1–7 days	48,545 (0.39%)	6,132 (0.45%)
ACSC admissions within 1–7 days	18,876 (0.15%)	1,989 (0.15%)
Referrals	534,873 (4.34%)	53,758 (3.93%)
Tests	395,795 (3.21%)	37,770 (2.76%)
**Means and standard deviations (sd) for patient and practice characteristics** **(*N* = 13,696,455 consultation events)**		
**Patient characteristics**		
Cambridge multi-morbidity score	0.46 (0.89)	0.37 (0.79)
Age	46 (23.7)	42 (23).1
Female	0.55 (0.50)	0.56 (0.50)
Years registered with the practice	16.4 (13.5)	14.8 (12.6)
Deprivation quintile	2.79 (1.4)	2.97 (1.41)
**Practice characteristics**		
Rurality	1.16 (0.37)	1.13 (0.33)
List size	7020 (3493)	6712 (3604)

**Table 2:** Descriptive statistics on potentially hazardous prescribing indicators, over 2010–2011 to 2021–2022

**Number of consultations at risk for potentially hazardous prescribing** **(*N* = 7,623,305 consultation events)**	*N*_*P*_ *Exposed to potentially hazardous prescribing, Permanent GP Numerator* *(% of denominator)*	*D*_*P*_ *At risk of potentially hazardous prescribing,* *Permanent GP Denominator*	*N*_*L*_ *Exposed to potentially hazardous prescribing,* *Locum GP* *Numerator* *(% of denominator)*	*D*_*L*_ *At risk of potentially hazardous prescribing,* *Locum GP* *Denominator*
**Potential harm: GI bleed**				
Prescription of an oral NSAID, without co-prescription of an ulcer healing drug, to a patient aged ≥ 65 years (*Indicator A*) (*N* = 454,929)	237,867 (56.5%)	420,760	19,926 (58.3%)	34,169
Prescription of an oral NSAID, without co-prescription of an ulcer healing drug, to a patient with a history of peptic ulceration (*Indicator B*) (*N* = 2907)	1376 (52.6%)	2617	171 (59%)	290
Prescription of an antiplatelet drug without co-prescription of an ulcer-healing drug, to a patient with a history of peptic ulceration (*Indicator C*) (*N* = 3537)	765 (23.1%)	3309	57 (27.4%)	228
Prescription of warfarin or DOAC in combination with an oral NSAID (*Indicator D*) (*N* = 222,986)	4567 (2.2%)	207,655	308 (2%)	15,331
Prescription of warfarin or DOAC and an antiplatelet drug in combination without co-prescription of an ulcer-healing drug (*Indicator E*) (*N* = 35,421)	15,856 (47.7%)	33,260	976 (45.2%)	2161
Prescription of aspirin in combination with another antiplatelet drug (without co-prescription of an ulcer-healing drug) (*Indicator F*) (*N* = 265,499)	124,950 (50.2%)	249,148	8328 (51%)	16,351
**Potential harm: exacerbation of asthma**				
Prescription of a non-selective beta-blocker to a patient with asthma (*Indicator G*) (*N* = 163,368)	11,183 (7.4%)	150,611	1165 (9.1%)	12,757
Prescription of a long-acting beta-2 agonist inhaler (excluding combination products with inhaled corticosteroid) to a patient with asthma who is not also prescribed an inhaled corticosteroid (*Indicator H*) (*N* = 5,917,201)	50,239 (0.09%)	5,407,846	3293 (0.06%)	509,355
**Potential harm: Heart failure**				
Prescription of an oral NSAID to a patient with heart failure (*Indicator I*) (*N* = 551,110)	2610 (0.05%)	551,110	195 (0.003%)	551,110
**Potential harm: Stroke**				
Prescription of antipsychotics for > 6 weeks in a patient aged ≥ 65 years with dementia but not psychosis (*Indicator J*) (*N* = 6347)	5601 (93.8%)	5972	335 (89.3%)	375

The results from our regression models are shown in Table 3. We found mixed differences between permanent and locum GPs in both the clinical practice indicators and the prescribing safety indicators, with some rates being higher or lower for locums and some non-significant differences.

**Table 3:** Mixed effects logistic regression for patient outcomes and potentially hazardous prescribing indicators over time, odds ratios

Effects of locum consultations on patient outcomes	
Practice revisits	0.88 (0.88 to 0.89), < 0.001 [0.003]
Antibiotic prescriptions	1.21 (1.21 to 1.22), < 0.001 [0.004]
Strong opioid prescriptions	1.08 (1.06 to 1.09), < 0.001 [0.007]
Hypnotic prescriptions	0.97 (0.95 to 0.99), < 0.002 [0.010]
Emergency admissions, same day	0.94 (0.89 to 1.02), < 0.127 [0.033]
Emergency admissions within 1 to 7 days	1.00 (0.96 to 1.06), < 0.854 [0.026]
A&E visits, same day	1.02 (0.98 to 1.07), < 0.331 [0.028]
A&E visits, within 1 to 7 days	1.05 (1.02 to 1.08), < 0.001 [0.015]
ACSC admissions, same day	1.00 (0.92 to 1.05), < 0.677 [0.033]
ACSC admissions, within 1 to 7 days	0.99 (0.94 to 1.04), < 0.890 [0.025]
Referrals	0.85 (0.84 to 0.86), < 0.001 [0.004]
Tests	0.80 (0.80 to 0.81), < 0.001 [0.005]
**Effects of locum consultations on potentially hazardous prescribing indicators**	
Indicator A	1.12 (1.08 to 1.16), < 0.001 [0.020]
Indicator B	1.44 (0.94 to 2.22), < 0.547 [0.331]
Indicator C	1.35 (0.72 to 2.54), < 0.349 [0.434]
Indicator D	0.77 (0.64 to 0.93), < 0.007 [0.074]
Indicator E	1.07 (0.79 to 1.44), < 0.675 [0.163]
Indicator F	0.99 (0.92 to 1.08), < 0.852 [0.042]
Indicator G	0.99 (0.91 to 1.09), < 0.894 [0.047]
Indicator H	0.89 (0.85 to 0.93), < 0.001 [0.021]
Indicator I	0.96 (0.81 to 1.13), < 0.613 [0.081]
Indicator J	0.49 (0.19 to 1.23), < 0.128 [0.230]

95% confidence intervals are in brackets; results are reported as incidence rate ratios (IRR) followed by *P*-values and standard errors in parentheses. Coefficients can be interpreted as proportionate changes, for example, patients who were seen by a locum GP were 12% less likely to revisit the practice within 7 days compared to patients who were seen by a permanent GP. Operational definitions of all indicators are provided in the Additional file 2: Table S3. Our regression analyses controlled for patient gender, age, comorbidity score, years registered with the practice, practice list size, the Index of Multiple Deprivation, region and year dummies

We find that a consultation with a locum was 12% less likely to lead to a practice revisit within 7 days (OR = 0.88, 95% CI 0.88 to 0.91). A consultation with a locum was 21% more likely to involve a prescription for an antibiotic (OR = 1.21, 95% CI 1.20 to 1.22), 8% more likely to involve a prescription for a strong opioid (OR = 1.08, 95% CI 1.06 to 1.09) and 3% less likely to involve a prescription for a hypnotic (OR = 0.97, 95% CI 0.94 to 0.99). Consultations with locums were also 15% less likely to involve a referral (OR = 0.85, 95% CI 0.84 to 0.86) and 20% less likely to involve a test being ordered (OR = 0.80, 95% CI 0.80 to 0.81). In terms of hospital-related outcomes, a consultation with a locum was 5% more likely to be followed by an A&E visit within 1 to 7 days (OR = 1.05, 95% CI 1.02 to 1.08) but there was no difference in rates of the same day A&E visits, emergency admissions or ACSC emergency admissions.

When comparing prescribing safety indicators for permanent and locum GPs, a consultation with a locum GP, was 11.2% (OR = 1.12, 95% CI 1.08 to 1.16) more likely to involve the prescription of an oral NSAID, without co-prescription of an ulcer healing drug, to a patient aged ≥ 65 years. But a consultation with a locum GP was 22.8% (OR = 0.77, 95% CI 0.64 to 0.93) less likely to involve the prescription of warfarin or a direct oral anticoagulant in combination with an oral NSAID, and 11.2% (OR = 0.89, 95% CI 0.85 to 0.93) less likely to involve the prescription of a long-acting beta-2 antagonist inhaler to a patient with asthma who is not also prescribed an inhaled corticosteroid. We didn’t find any significant differences between permanent and locum GPs across all other prescribing safety indicators. The full output from the multilevel regressions is presented in Table S18–S22 in Additional file 2.

**Fig. 1:**
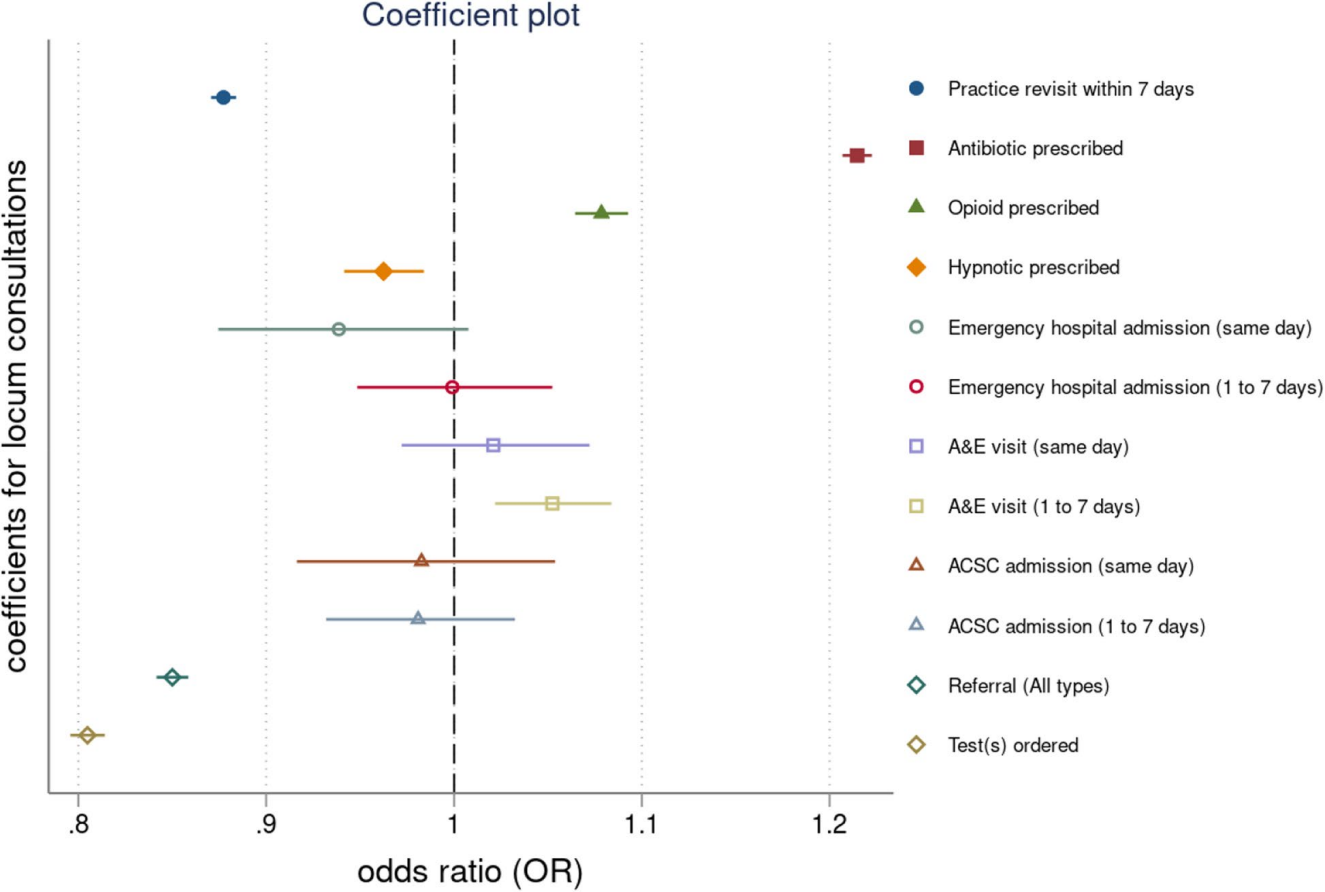
Coefficient plot for locum consultations across all outcomes. Note: Results are expressed as odd ratios (OR) and corresponding confidence intervals (CI). When the corresponding CIs cross the dashed vertical line coefficients are not statistically significant

**Fig. 2:**
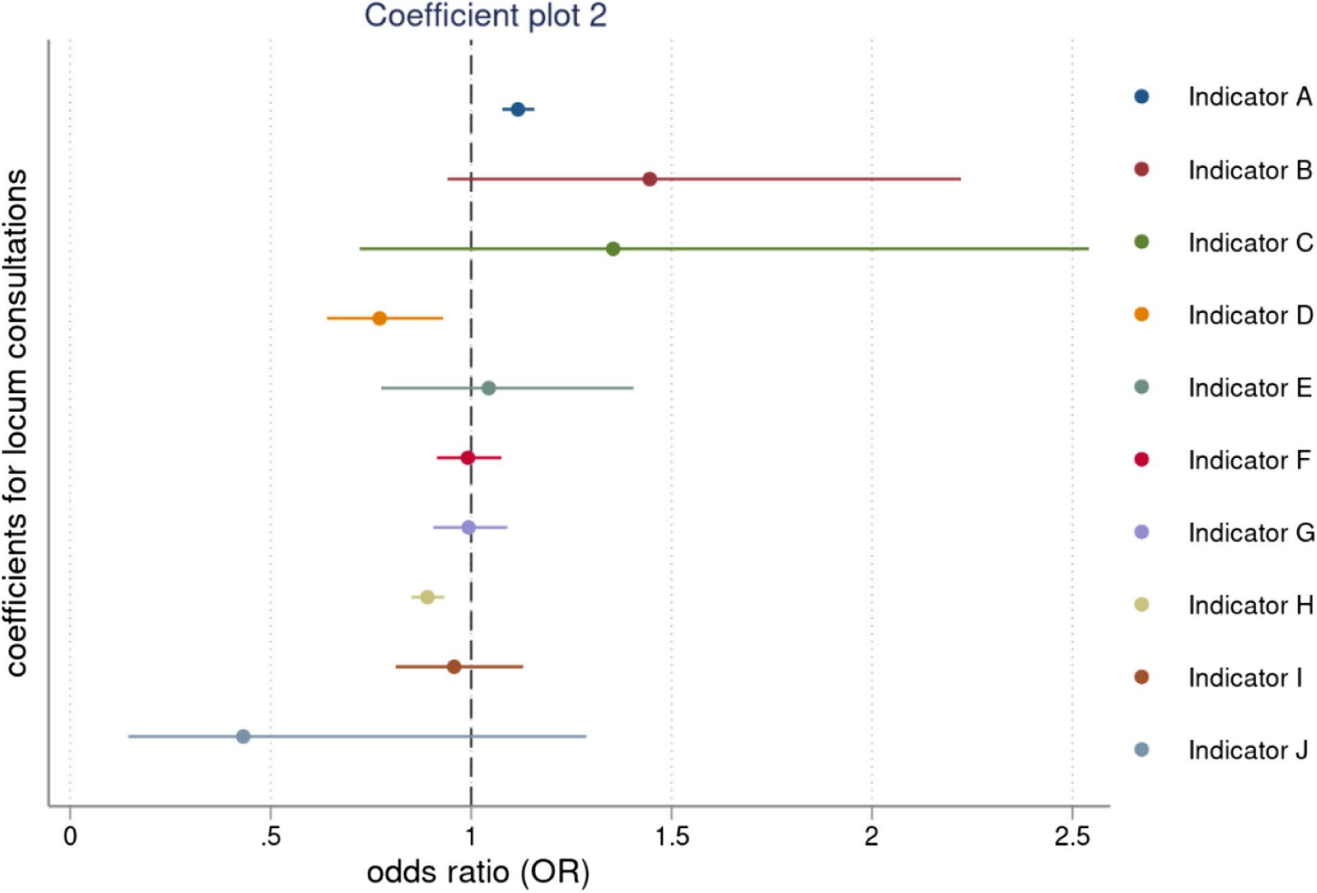
Coefficient plot for locum consultations across prescribing safety indicators. Note: Results are expressed as odd ratios (OR) and corresponding confidence intervals (CI). When the corresponding CIs cross the dashed vertical line coefficients are not statistically significant

We plotted the effects and the confidence intervals of locum consultations on the patient outcomes in Fig. 1 and the effects and confidence intervals of locum consultation on the potentially hazardous prescribing indicators in Fig. 2. The results from the sensitivity analyses, excluding the period 2020–2022, were effectively the same and we report them in Table S23 in Additional file 2.

## Discussion

This study set out to examine differences in the clinical practice and consultation outcomes for locum and permanent doctors working in primary care in England. Our findings suggest there are some differences, but their interpretation is complex and should be approached with caution.

For example, when considering a return to practice within 7 days, we find that this is 12% less likely for a locum GP than for a permanent GP. To some, this may seem counter-intuitive as they might have expected that patients seeing a locum GP could be less satisfied or assured by the consultation and so more likely to return within a week. But there are some important other considerations to take into account. Patients who prefer not to see a locum may have opted to wait to see a permanent GP, so the patient groups may not be comparable. Some practices may assign more straightforward cases to locums and although our regression models adjust for comorbidities, there are probably unmeasured differences in the case mix characteristics of patients seeing locum and permanent GPs. Moreover, our qualitative research suggests that some patients actually welcome the opportunity to see a locum GP because they get a fresh perspective on their condition [36].

However, we also find that locums consistently prescribed antibiotics and strong opioids more than permanent GPs, and we might speculate that locums could be less aware of or compliant with practice prescribing guidelines, or that locums may be more inclined to respond to patient requests for a prescription [36]. Other research has also suggested that locum doctors prescribe antibiotics more readily [37].

Perhaps our most striking finding is that locum GPs are markedly less likely to both order tests and refer patients to other services (such as hospital outpatient clinics) than permanent GPs. But here, we suspect this may in part be because practices set constraints on such decisions by locums, requiring them to be reviewed or approved by another GP in the practice [36].

To put these differences in outcomes into context, it can be helpful to consider how commonly they occur, which is reported in Table 1. Several of the outcomes where differences are observed are also common: practice revisits (8%), antibiotic prescription (9%), referral (4%) and test (3%). However, other outcomes are comparatively rare: strong opioid prescription (2%), hypnotic prescription (< 1%), and A&E visit (0.15%). Therefore, our findings relating to revisits, antibiotics, referrals and tests are both statistically and clinically significant.

On the PINCER prescribing safety indicators, again the results are quite mixed. On most indicators, there is no significant difference between locum and permanent GPs — and the differences we do observe on three indicators are not large and move in different directions. There certainly seems to be no basis to argue that locum GPs differ significantly from permanent GPs on these indicators.

Past research largely in inpatient acute care has also found mixed differences in care between locum and permanent doctors. A US study investigating the impact of locum working on patient outcomes, including mortality, 30-day hospital readmissions and cost of care found significant differences in mortality rates for patients who were treated by locums who had worked for less than 60 days in the organisation but no significant differences for patients who were treated by locums who had worked in the organisation for 60 days or more [7]. Another study comparing locum and permanent doctors found locum doctors had shorter stays and lower treatment costs but there were no differences in mortality or readmissions [38].

### Strengths and limitations of the study

This is the largest observational study of locum and permanent doctor consultations in primary care investigating differences in clinical practice indicators and prescribing safety indicators. However, there are important limitations. First, any study of this nature is limited by the reliability and accuracy of the data in the patient’s electronic record. We are confident about the reliability of the recorded patient contact data and patient characteristics as consultation events are central to how CPRD GOLD is organised but we could not assess the reliability with which the staff role field linked to each consultation is recorded. Moreover, we know from other research [39] that locum doctors working in primary care may undertake anything from very short placements of a few days in a practice to very much longer or regular placements as the preferred locum for a practice — and we could not distinguish between such short-term and long-term locums in our analysis. It may also be that some long-term and regular locum GPs working in practices get recorded on the Vision system as permanent GPs. If GP identifiers were made available this could be considered in future research.

Second, we were not able to assess the reasons why patients revisited their general practice within 7 days following a consultation with a locum GP and to distinguish for example between planned follow-ups from the first consultation and unplanned revisits, or between revisits which were clinically related to the first consultation and those which were unrelated and for another matter. Third, there may be other systemic differences between locum and permanent GPs which are not available within CPRD but which might be material to our analysis — such as gender, age, ethnicity, years of experience as a GP, where they qualified and trained, and so on. Additional information on locum doctor working arrangements as well as demographic information about doctors would enable more detailed comparisons between locum and permanent GPs [40]. Fourth, CPRD GOLD is representative of the UK population in terms of deprivation and population characteristics [15], but data is collected from a single clinical information system (Vision) and contributing practices are not uniformly distributed across English regions, while its market share is in decline [18]. Thus, generalisability to every English region could not be achieved.

## Conclusions

We noted earlier that locum doctors are often regarded with some suspicion and portrayed by some as less clinically competent or professionally committed than permanent doctors [12] but there is little evidence in our findings to suggest that systemic differences exist in practice or performance between locum and permanent GPs. Rather, it seems likely that the performance of both locum and permanent GPs is shaped by the wider organisational context in which they practice — the quality of induction, supervision, communication, and practice management being obvious likely determinants [8]. Locums form a necessary component of the overall medical workforce and can enable practices to cope with staff shortages, planned or unplanned staff absences and variations in demand for appointments. Future research should focus on understanding how organisations can make the best use of locums as part of their wider medical workforce and how locum doctors can be enabled to practice and perform effectively as members of the clinical team.

### Supplementary Information

**Additional file 1:** **Table S1.** Codelist for antibiotics. **Table S2.** Codelist for opioids. **Table S3.** Codelist for benzodiazepines. **Table S4.** Codelist for z-drugs. **Table S5.** Codelist for conditions linked to potentially hazardous prescribing (READ codes). **Table S6.** Codelist for anticoagulants. **Table S7.** Codelist for antipsychotic drugs. **Table S8.** Codelist for aspirin products. **Table S9.** Codelist for antiplatelet drugs (non-aspirin). **Table S10.** Codelist for β-blockers. **Table S11.** Codelist for inhaler corticosteroids. **Table S12.** Codelist for long-acting beta-2 antagonists. **Table S13.** Codelist for non-steroidal anti-inflammatory drugs (NSAIDS). **Table S14.** Codelist for non-selective β-blockers. **Table S15.** Codelist for proton-pump inhibitors (PPIs) and H2 blockers.

**Additional file 2:** **Table S16.** ICD-10 Codes for hospital admissions. **Table S17.** Definitions for PINCER Indicators. **Table S18.** Regression analyses for patient outcomes, pt.1. **Table S19.** Regression analyses for patient outcomes, pt.2. **Table S20.** Regression analyses for patient outcomes, pt.3. **Table S21.** Regression analyses for prescribing safety outcomes, pt.1. **Table S22.** Regression analyses for prescribing safety outcomes, pt.2. **Table S23.** Regression analyses for prescribing safety outcomes, excluding 2020–2022.

## Data Availability

In this study, we used anonymised patient-level data from the CPRD that are not publicly available due to confidentiality considerations. However, researchers can access CPRD’s databases by contacting the CPRD. Details of the application process and conditions of access are available at https://www.cprd.com/Data-access.

## Abbreviations

A&E: Accident and emergency
ACSCs: Ambulatory care sensitive conditions
BNF: British National Formulary
CI: Confidence interval
CPRD: Clinical Practice Research Datalink
DOAC: Direct oral anticoagulants
EHRs: Electronic health records
GP: General practitioner
HES: Hospital Episode Statistics
ICD-10: International Classification of Disease
IMD: Index of Multiple Deprivation
LSOA: Lower Layer Super Output Area
MHRA: Medicines & Healthcare Products Regulatory Agency
NHS: National Health Service
NSAID: No-steroidal anti-inflammatory drugs
OR: Odds ratio
PINCER: Pharmacist-led information technology intervention
PPI: Proton-pump inhibitor
QOF: Quality and Outcomes Framework
UK: United Kingdom
