# Supplementary material for: Comparing the clinical practice and prescribing safety of locum and permanent doctors: observational study of primary care consultations in England
Source: BMC Med. 2024 Mar 27;22:126. doi: 10.1186/s12916-024-03332-z (PMC10967114; doi:10.1186/s12916-024-03332-z)
Supplement: Supplementary file 2 — Additional file 2: Table S16. ICD-10 Codes for hospital admissions. Table S17. Definitions for PINCER Indicators. Table S18. Regression analyses for patient outcomes, pt.1. Table S19. Regression analyses for patient outcomes, pt.2. Table S20. Regression analyses for patient outcomes, pt.3. Table S21. Regression analyses for prescribing safety outcomes, pt.1. Table S22. Regression analyses for prescribing safety outcomes, pt.2. Table S23. Regression analyses for prescribing safety outcomes, excluding 2020–2022. [file 12916_2024_3332_MOESM2_ESM.docx]

**Additional file 2 - Comparing the clinical practice and prescribing safety of locum and permanent doctors: observational study of primary care consultations in England.**

Contents

[Table S16 – ICD-10 codes used to define emergency admission and ACSCs 3](#_Toc159861183)

[Table S17 – Operational definitions for hazardous prescribing indicators A-J (PINCER) 4](#_Toc159861184)

[Table S18 - Mixed effects logistic regression for patient outcomes (4/12) over time pt1, 7](#_Toc159861185)

[Table S19 - Mixed effects logistic regression for patient outcomes (8/12) over time pt2, 9](#_Toc159861186)

[Table S20 - Mixed effects logistic regression for patient outcomes (12/12) over time pt3, 11](#_Toc159861187)

[Table S21 - Mixed effects logistic regression for safety indicators (5/10) over time pt1, 13](#_Toc159861188)

[Table S22 - Mixed effects logistic regression for safety indicators (10/10) over time pt2, 15](#_Toc159861189)

[Table S23 Mixed effects logistic regression for patient outcomes and potentially hazardous prescribing indicators over time (excluding 2020-2022), 17](#_Toc159861190)

## Table S16 – ICD-10 codes used to define emergency admission and ACSCs

| **ACSC Chapter (QOF incentivised)** | **ICD-10 chapter (QOF incentivised)** |
| --- | --- |
| Asthma | J45, J46 |
| Coronary Heart Disease | I20, I24.0, I24.8, I24.9, I25 |
| Chronic Obstructive Pulmonary Disease | J20, J41, J42, J43, J44, J47 |
| Diabetes | E10.0–E10.8, E11.0–E11.8, E13.0–E13.8, E14.0–E14.8 |
| Dementia | I48, F00, F01, F02, F03 |
| Epilepsy | G40, G41 |
| Heart Failure | I11.0, I13.0, I50, J81 |
| Hypertension | I10, I11.9 |
| Stroke | I61, I62, I63, I64, I66, I67.2, I69.8, R47.0 |

## Table S17 – Operational definitions for hazardous prescribing indicators A-J (PINCER)

| **QUERY** | **DESCRIPTION OF INDICATOR** | **GROUP AT RISK**  **(DENOMINATOR)** | **GROUP EXPOSED TO HAZARDOUS PRESCRIBING (NUMERATOR)** | **OPERATIONALISATION OF HAZARDOUS PRESCRIBING INDICATORS FOR LOCUM AND PERMANENT GPs.** |
| --- | --- | --- | --- | --- |
|  | OUTCOME: GI BLEED | | |  |
| **A** | Prescription of an oral NSAID, without co-prescription of an ulcer healing drug, to a patient aged ≥65 years | Patients aged ≥65 years without co-prescription of an ulcer-healing drug (PPI or H_2_ antagonist) in the 3 months leading up to the index date | Patients prescribed an oral NSAID in the 3 months leading up to the index date | 1. Identify patients over 65 prescribed an NSAID. This is the denominator. Split by locum [D^L^] and permanent GP [D^P^]. 2. For both D. Identify those patients who were not prescribed PPI or H_2_ antagonist 3 months before NSAID. This is the numerator i.e. determines if the NSAID was hazardous [N^L^ and N^P^]. |
| **B** | Prescription of an oral NSAID, without co-prescription of an ulcer healing drug, to a patient with a history of peptic ulceration | Patients aged ≥18 years with a Read code for peptic ulcer or upper GI bleed at least 3 months before index date and not prescribed an ulcer healing drug (PPI or H_2_ antagonist) within the 3 months leading up to the index date | Patients prescribed an oral NSAID within the 3 months leading up to the index date | 1. Identify patients over 18 and Read code for peptic ulcer or upper GI bleed [read codes identified in the 24-month period prior to the index consultation event]. 2. From #1 find those prescribed an NSAID. This is the denominator. Split by locum [D^L^] and permanent GP [D^P^]. 3. Identify those not prescribed PPI or H_2_ antagonist 3 months before NSAID. This is the numerator. |
| **C** | Prescription of an antiplatelet drug without co-prescription of an ulcer-healing drug, to a patient with a history of peptic ulceration | Patients aged ≥18 years with a Read code for peptic ulcer or GI bleed at least 3 months before index date and not prescribed an ulcer healing drug (PPI or H_2_ antagonist) within the 3 months leading up to the index date | Patients prescribed an antiplatelet drug (aspirin or clopidogrel or prasugrel or ticagrelor) within the 3 months leading up to the index date | 1. Identify patients over 18 and Read code for peptic ulcer or upper GI bleed [read codes identified in the 24-month period prior to the index consultation event]. 2. From #1 find those prescribed an antiplatelet. This is the denominator. Split by locum [D^L^] and permanent GP [D^P^]. 3. Identify those not prescribed PPI or H_2_ antagonist 3 months before antiplatelet drug. This is the numerator. |
| **D** | Prescription of warfarin or DOAC in combination with an oral NSAID | Patients aged ≥18 years prescribed warfarin or a DOAC (apixaban or dabigatran or rivaroxaban) within the 3 months leading up to the index date | Patients prescribed an oral NSAID within the 3 months leading up to the index date | 1. Identify patients over 18 and prescribed an NSAID. This is the denominator. Split by locum [D^L^] and permanent GP [D^P^]. 2. Identify those prescribed warfarin/DOAC in 3 months before NSAID. This is the numerator. |
| **E** | Prescription of warfarin or DOAC and an antiplatelet drug in combination without co-prescription of an ulcer-healing drug | Patients aged ≥18 years prescribed warfarin or DOAC without co-prescription of ulcer-healing drug (PPI or H_2_ antagonist) within the 3 months leading up to the index date | Patients prescribed an antiplatelet drug (aspirin or clopidogrel or prasugrel or ticagrelor) within the 3 months leading up to the index date and within 28 days of the warfarin/DOAC prescription | 1. Identify patients over 18 prescribed warfarin or DOAC and prescribed an antiplatelet within 28 days or the warfarin/DOAC prescription. This is the denominator. Split by locum [D^L^] and permanent GP [D^P^]. 2. For both D. Identify those not prescribed PPI or H_2_ antagonist 3 months before antiplatelet. This is the numerator i.e. determines if the antiplatelet was hazardous [N^L^ and N^P^]. |
| **F** | Prescription of aspirin in combination with another antiplatelet drug (without co-prescription of an ulcer-healing drug) | Patients aged ≥18 years prescribed aspirin without co-prescription of ulcer-healing drug (PPI or H_2_ antagonist) within the 3 months leading up to the index date | Patients prescribed another antiplatelet drug (clopidogrel or prasugrel or ticagrelor) within the 3 months leading up to the index date and within 28 days of the aspirin prescription | 1. Identify over 18 prescribed aspirin and prescribed an antiplatelet within 28 days or the aspirin prescription. This is the denominator. Split by locum [D^L^] and permanent GP [D^P^]. 2. For both D. Identify those not prescribed PPI or H_2_ antagonist 3 months before antiplatelet. This is the numerator i.e. determines if the antiplatelet was hazardous [N^L^ and N^P^]. |
|  | OUTCOME: EXACERBATION OF ASTHMA | | |  |
| **G** | Prescription of a non-selective beta-blocker to a patient with asthma | Patients aged ≥18 years with a Read code for asthma at least 3 months before index date and no subsequent asthma resolved code during that time period | Patients prescribed a non-selective β-blocker within the 3 months leading up to the index date | 1. Identify patients over 18 with a Read code for asthma and prescribed a selective β-blocker. This is the denominator. Split by locum [D^L^] and permanent GP [D^P^]. 2. For both D. Identify those prescribed a non-selective β-blocker 3 months before selective β-blocker. This is the numerator i.e. determines if the selective β-blocker was hazardous [N^L^ and N^P^]. |
| **H** | Prescription of a long-acting beta-2 agonist inhaler (excluding combination products with inhaled corticosteroid) to a patient with asthma who is not also prescribed an inhaled corticosteroid | Patients aged ≥18 years with a Read code for asthma at least 3 months before index date (and no subsequent asthma resolved code during that time period) who have been prescribed a long acting beta-2 agonist inhaler (excluding combination products with inhaled corticosteroid) within the last 3 months | Patients not prescribed an inhaled corticosteroid within the 3 months leading up to the index date | 1. Identify patients over 18 with a Read code for asthma and not prescribed an inhaled corticosteroid. This is the denominator. Split by locum [D^L^] and permanent GP [D^P^]. 2. For both D. Identify those prescribed a long-lasting beta-2 agonist inhaler. This is the numerator [N^L^ and N^P^]. |
|  | OUTCOME: HEART FAILURE | | |  |
| **I** | Prescription of an oral NSAID to a patient with heart failure | Patients aged ≥18 years who have a diagnosis of heart failure at least 3 months before the index date | Patients prescribed an oral NSAID within the 3 months leading up to the index date | 1. Identify over 18 with a Read code for heart failure. This is the denominator. This is the same denominator for everyone [D^i^]. 2. For both D. Identify those prescribed an NSAID. This is the numerator i.e. determines if the NSAID was hazardous. [N^L^ and N^P^]. |
|  | OUTCOME: CARDIOVASCULAR EVENTS, INCLUDING STROKE | | |  |
| **J** | Prescription of antipsychotics for >6weeks in a patient aged ≥65 years with dementia but not psychosis | Patients aged ≥65 years with a Read code for dementia at least 3 months before the index date and no Read code for psychosis (or have a psychosis Read code and a subsequent psychosis resolved Read code) at least 3 months before the index date | Patients prescribed antipsychotic drugs at least once within the 3 months leading up to the index date | 1. Identify over 65 with a Read code for dementia an antipsychotic drug. This is the denominator. Split by locum [D^L^] and permanent GP [D^P^]. 2. For both D. Identify those with no Read code for psychosis. This is the numerator i.e. determines if the antipsychotic drug was hazardous [N^L^ and N^P^]. |

| Table S18 - Mixed effects logistic regression for patient outcomes (4/12) over time pt1, OR a,b | | | | |
| --- | --- | --- | --- | --- |
|  | Practice revisits | Antibiotic prescriptions | Opioid prescriptions | Hypnotic prescriptions |
| Locum consultations | **0.884 (0.878 to 0.891),<0.001[0.003]** | **1.213 (1.207 to 1.220),<0.001[0.004]** | **1.077 (1.063 to 1.091),<0.001[0.007]** | **0.967 (0.946 to 0.988),<0.002[0.010]** |
| Gender (reference= male) | 1.177 (1.172 to 1.181),<0.001[0.002] | 1.107 (1.103 to 1.112),<0.001[0.002] | 1.044 (1.037 to 1.052),<0.001[0.003] | 1.179 (1.166 to 1.194),<0.001[0.007] |
| Age | 1.005 (1.004 to 1.005),<0.001[0.001] | 0.988 (0.988 to 0.989),<0.001[0.001] | 1.018 (1.018 to 1.018),<0.001[0.001] | 1.012 (1.012 to 1.013),<0.001[0.001] |
| Comorbidity score | 1.197 (1.194 to 1.199),<0.001[0.001] | 1.016 (1.013 to 1.019),<0.001[0.001] | 1.033 (1.029 to 1.037),<0.001[0.001] | 0.998 (0.991 to 1.004),<0.538[0.003] |
| Years registered  with practice | 0.998 (0.997 to 0.998),<0.001[0.001] | 1.001 (1.001 to 1.002),<0.001[0.001] | 0.998 (0.998 to 0.999),<0.001[0.001] | 0.994 (0.994 to 0.995),<0.001[0.001] |
| Patient list size | 1.001 (1.000 to 1.001),<0.001[0.001] | 1.000 (1.000 to 1.001),<0.001[0.001] | 0.999 (0.999 to 1.000),<0.028[0.001] | 0.999 (0.999 to 0.999),<0.001[0.001] |
| IMD quintile  (1=most deprived) |  | | | |
| Quintile 2 | 1.016 (1.009 to 1.022),<0.001[0.003] | 1.009 (1.003 to 1.015),<0.005[0.003] | 1.161 (1.146 to 1.176),<0.001[0.007] | 1.028 (1.008 to 1.048),<0.004[0.009] |
| Quintile 3 | 1.027 (1.020 to 1.034),<0.001[0.003] | 1.000 (0.994 to 1.006),<0.886[0.003] | 1.286 (1.270 to 1.303),<0.001[0.008] | 1.086 (1.065 to 1.107),<0.001[0.010] |
| Quintile 4 | 1.049 (1.041 to 1.057),<0.001[0.004] | 0.992 (0.985 to 0.999),<0.027[0.003] | 1.451 (1.431 to 1.470),<0.001[0.010] | 1.193 (1.168 to 1.219),<0.001[0.012] |
| Quintile 5 | 1.057 (1.049 to 1.066),<0.001[0.004] | 0.977 (0.970 to 0.984),<0.001[0.003] | 1.702 (1.678 to 1.727),<0.001[0.011] | 1.336 (1.305 to 1.368),<0.001[0.016] |
| Rurality | 1.002 (0.993 to 1.011),<0.683[0.004] | 0.991 (0.992 to 1.008),<0.976[0.004] | 0.925 (0.910 to 0.941),<0.001[0.007] | 0.972 (0.948 to 0.997),<0.026[0.012] |
| Region  (reference=N. East) |  | | | |
| North West | 0.863 (0.704 to 1.057),<0.155[0.090] | 1.201 (0.989 to 1.458),<0.064[0.119] | 0.982 (0.822 to 1.173),<0.839[0.089] | 1.214 (0.930 to 1.584),<0.153[0.165] |
| Yorkshire & Humber | 0.935 (0.728 to 1.200),<0.596[0.120] | 1.107 (0.873 to 1.405),<0.400[0.134] | 0.961 (0.773 to 1.194),<0.717[0.107] | 1.020 (0.736 to 1.414),<0.906[0.170] |
| Midlands | 0.872 (0.709 to 1.073),<0.196[0.088] | 1.099 (0.903 to 1.339),<0.346[0.111] | 0.940 (0.785 to 1.126),<0.501[0.086] | 1.323 (1.011 to 1.735),<0.042[0.182] |
| East of England | 0.945 (0.763 to 1.169),<0.601[0.104] | 1.075 (0.877 to 1.317),<0.484[0.111] | 0.857 (0.711 to 1.032),<0.103[0.081] | 1.562 (1.182 to 2.063),<0.002[0.222] |
| London | 0.861 (0.703 to 1.055),<0.150[0.090] | 1.086 (0.895 to 1.318),<0.401[0.107] | 0.693 (0.580 to 0.827),<0.001[0.063] | 1.228 (0.942 to 1.602),<0.129[0.165] |
| South East | 0.950 (0.778 to 1.162),<0.620[0.099] | 1.154 (0.953 to 1.396),<0.143[0.112] | 0.840 (0.705 to 1.001),<0.050[0.075] | 1.434 (1.104 to 1.863),<0.007[0.191] |
| South West | 1.110 (0.901 to 1.368),<0.326[0.119] | 1.035 (0.849 to 1.262),<0.733[0.105] | 0.869 (0.724 to 1.042),<0.129[0.081] | 1.517 (1.155 to 1.991),<0.003[0.211] |
| Year (reference year=2010) |  | | | |
| 2011 | 1.043 (1.035 to 1.050),<0.001[0.003] | 0.946 (0.940 to 0.952),<0.001[0.003] | 1.004 (0.992 to 1.017),<0.490[0.006] | 0.974 (0.954 to 0.994),<0.011[0.010] |
| 2012 | 1.079 (1.071 to 1.087),<0.001[0.004] | 0.973 (0.967 to 0.980),<0.001[0.003] | 0.964 (0.952 to 0.977),<0.001[0.006] | 0.910 (0.891 to 0.929),<0.001[0.009] |
| 2013 | 1.098 (1.089 to 1.106),<0.001[0.004] | 0.864 (0.859 to 0.870),<0.001[0.003] | 0.962 (0.950 to 0.976),<0.001[0.007] | 0.913 (0.893 to 0.932),<0.001[0.010] |
| 2014 | 1.111 (1.103 to 1.121),<0.001[0.004] | 0.859 (0.853 to 0.866),<0.001[0.003] | 0.944 (0.930 to 0.958),<0.001[0.007] | 0.886 (0.866 to 0.907),<0.001[0.010] |
| 2015 | 1.116 (1.106 to 1.149),<0.001[0.005] | 0.758 (0.752 to 0.764),<0.001[0.003] | 0.941 (0.926 to 0.955),<0.001[0.008] | 0.866 (0.845 to 0.889,<0.001[0.011] |
| 2016 | 1.138 (1.127 to 1.155),<0.001[0.005] | 0.731 (0.723 to 0.737),<0.001[0.003] | 0.907 (0.890 to 0.923),<0.001[0.008] | 0.848 (0.823 to 0.872),<0.001[0.012] |
| 2017 | 1.143 (1.131 to 1.135),<0.001[0.006] | 0.720 (0.713 to 0.728),<0.001[0.004] | 0.857 (0.840 to 0.875),<0.001[0.009] | 0.837 (0.811 to 0.865),<0.001[0.013] |
| 2018 | 1.122 (1.109 to 1.141),<0.001[0.006] | 0.668 (0.660 to 0.675),<0.001[0.004] | 0.805 (0.787 to 0.823),<0.001[0.009] | 0.798 (0.770 to 0.826),<0.001[0.013] |
| 2019 | 1.127 (1.113 to 1.141),<0.001[0.007] | 0.658 (0.648 to 0.664),<0.001[0.004] | 0.760 (0.742 to 0.779),<0.001[0.009] | 0.743 (0.714 to 0.773),<0.001[0.013] |
| 2020 | 1.483 (1.462 to 1.505),<0.001[0.010] | 0.625 (0.614 to 0.636),<0.001[0.005] | 0.773 (0.749 to 0.797),<0.001[0.011] | 0.734 (0.697 to 0.774),<0.001[0.017] |
| 2021 | 1.488 (1.460 to 1.516),<0.001[0.014] | 0.633 (0.618 to 0.648),<0.001[0.007] | 0.748 (0.717 to 0.780),<0.001[0.015] | 0.660 (0.611 to 0.714),<0.001[0.023] |
| constant | 0.052 (0.043 to 0.064),<0.001[0.005] | 0.148 (0.123 to 0.178),<0.001[0.014] | 0.009 (0.007 to 0.010),<0.001[0.001] | 0.004 (0.002 to 0.005),<0.001[0.001] |

a 95% confidence intervals are in brackets; results are reported as incidence rate ratios (IRR) followed by P-values and standard errors in parentheses.

b Coefficients can be interpreted as proportionate changes, for example, patients in the North West had on average 20.1% more antibiotic prescriptions than .patients in the North East

| Table S19 - Mixed effects logistic regression for patient outcomes (8/12) over time pt2, OR a,b | | | | |  |
| --- | --- | --- | --- | --- | --- |
|  | **Emergency admissions,**  **same day** | **Emergency admissions,**  **within 1 to 7 days** | **A&E visits,**  **same day** | **A&E visits,**  **within 1 to 7 days** | |
| **Locum consultations** | **0.942 (0.887 to 1.015),<0.127[0.033]** | **0.999 (0.956 to 1.056),<0.854[0.026]** | **1.023 (0.977 to 1.073),<0.331[0.028]** | **1.052 (1.022 to 1.082),<0.001[0.015]** | |
| **Gender (reference= male)** | 0.921 (0.888 to 0.956),<0.001[0.017] | 0.911 (0.887 to 0.937),<0.001[0.012] | 0.892 (0.869 to 0.918),<0.001[0.012] | 0.870 (0.856 to 0.885),<0.001[0.007] | |
| **Age** | 1.011 (1.010 to 1.012),<0.001[0.001] | 1.008 (1.007 to 1.009),<0.001[0.001] | 0.988 (0.988 to 0.989),<0.001[0.001] | 0.987 (0.986 to 0.987),<0.001[0.001] | |
| **Comorbidity score** | 1.373 (1.353 to 1.394),<0.001[0.010] | 1.370 (1.354 to 1.386),<0.001[0.001] | 1.262 (1.243 to 1.282),<0.001[0.010] | 1.190 (1.178 to 1.203),<0.001[0.006] | |
| **Years registered**  **with practice** | 1.001 (1.000 to 1.002),<0.172[0.001] | 0.998 (0.997 to 0.999),<0.001[0.001] | 0.997 (0.995 to 0.998),<0.001[0.001] | 0.997 (0.997 to 0.998),<0.001[0.001] | |
| **Patient list size** | 0.999 (0.999 to 1.001),<0.114[0.001] | 0.999 (0.998 to 0.999),<0.006[0.001] | 1.000 (0.999 to 1.000),<0.001[0.001] | 0.999 (0.998 to 0.999),<0.001[0.001] | |
| **IMD quintile**  **(1=most deprived)** |  | | | | |
| **Quintile 2** | 1.148 (1.080 to 1.107),<0.001[0.035] | 1.098 (1.049 to 1.150),<0.001[0.025] | 1.112 (1.062 to 1.166),<0.001[0.027] | 1.096 (1.064 to 1.127),<0.001[0.016] | |
| **Quintile 3** | 1.180 (1.108 to 1.169),<0.001[0.038] | 1.192 (1.137 to 1.249),<0.001[0.028] | 1.194 (1.137 to 1.253),<0.001[0.030] | 1.123 (1.091 to 1.157),<0.001[0.017] | |
| **Quintile 4** | 1.318 (1.234 to 1.224),<0.001[0.044] | 1.257 (1.196 to 1.321),<0.001[0.030] | 1.256 (1.195 to 1.321),<0.001[0.033] | 1.211 (1.174 to 1.250),<0.001[0.019] | |
| **Quintile 5** | 1.414 (1.318 to 1.283),<0.001[0.051] | 1.336 (1.266 to 1.410),<0.001[0.035] | 1.371 (1.299 to 1.447),<0.001[0.038] | 1.268 (1.225 to 1.311),<0.001[0.022] | |
| **Rurality** | 0.955 (0.892 to 1.049),<0.195[0.034] | 0.927 (0.877 to 0.979),<0.005[0.025] | 0.929 (0.876 to 0.984),<0.001[0.028] | 0.945 (0.910 to 0.981),<0.003[0.018] | |
| **Region**  **(reference=N. East)** |  | | | | |
| **North West** | 0.973 (0.765 to 1.238),<0.824[0.119] | 0.995 (0.805 to 1.228),<0.961[0.107] | 1.110 (0.885 to 1.390),<0.367[0.127] | 1.354 (1.103 to 1.663),<0.004[0.142] | |
| **Yorkshire & Humber** | 0.824 (0.604 to 1.122),<0.219[0.130] | 0.986 (0.756 to 1.285),<0.917[0.133] | 0.818 (0.612 to 1.092),<0.173[0.121] | 1.104 (0.856 to 1.424),<0.444[0.143] | |
| **Midlands** | 1.126 (0.882 to 1.439),<0.340[0.141] | 1.143 (0.922 to 1.417),<0.222[0.125] | 1.216 (0.966 to 1.530),<0.095[0.143] | 1.357 (1.101 to 1.672),<0.004[0.145] | |
| **East of England** | 1.038 (0.805 to 1.338),<0.773[0.135] | 1.010 (0.808 to 1.261),<0.933[0.114] | 1.061 (0.836 to 1.346),<0.627[0.129] | 1.219 (0.982 to 2.511),<0.072[0.134] | |
| **London** | 1.008 (0.791 to 1.284),<0.948[0.125] | 0.930 (0.753 to 1.150),<0.505[0.099] | 1.467 (1.170 to 1.837),<0.001[0.169] | 1.575 (1.283 to 1.933),<0.001[0.165] | |
| **South East** | 0.974 (0.768 to 1.235),<0.826[0.118] | 0.925 (0.751 to 1.139),<0.461[0.098] | 1.090 (0.873 to 1.362),<0.446[0.124] | 1.248 (1.020 to 1.529),<0.032[0.129] | |
| **South West** | 0.890 (0.694 to 1.141),<0.357[0.113] | 0.853 (0.686 to 1.060),<0.152[0.094] | 0.987 (0.782 to 1.245),<0.910[0.117] | 1.170 (0.947 to 1.444),<0.145[0.126] | |
| **Year (reference year=2010)** |  | | | | |
| **2011** | 0.998 (0.932 to 1.069),<0.959[0.035] | 1.075 (1.132 to 1.132),<0.005[0.028] | 1.107 (1.051 to 1.167),<0.001[0.029] | 1.142 (1.106 to 1.179),<0.001[0.019] | |
| **2012** | 0.997 (0.931 to 1.069),<0.941[0.035] | 1.083 (1.141 to 1.141),<0.001[0.029] | 1.148 (1.089 to 1.209),<0.001[0.031] | 1.187 (1.150 to 1.225),<0.001[0.019] | |
| **2013** | 1.041 (0.970 to 1.116),<0.270[0.037] | 1.057 (1.115 to 1.115),<0.014[0.029] | 1.139 (1.080 to 1.202),<0.001[0.031] | 1.191 (1.154 to 1.231),<0.014[0.020] | |
| **2014** | 1.031 (0.958 to 1.109),<0.421[0.039] | 1.090 (1.153 to 1.153),<0.005[0.031] | 1.208 (1.143 to 1.277),<0.001[0.034] | 1.222 (1.182 to 1.265),<0.005[0.021] | |
| **2015** | 1.052 (0.972 to 1.138),<0.208[0.042] | 1.091 (1.158 to 1.158),<0.009[0.033] | 1.233 (1.162 to 1.309),<0.001[0.037] | 1.240 (1.195 to 1.285),<0.009[0.023] | |
| **2016** | 1.109 (1.016 to 1.211),<0.021[0.050] | 1.118 (1.197 to 1.197),<0.001[0.039] | 1.210 (1.131 to 1.294),<0.001[0.041] | 1.211 (1.162 to 1.262),<0.001[0.026] | |
| **2017** | 1.152 (1.047 to 1.269),<0.004[0.057] | 1.170 (1.260 to 1.260),<0.001[0.044] | 1.198 (1.113 to 1.290),<0.001[0.045] | 1.250 (1.195 to 1.307),<0.001[0.029] | |
| **2018** | 1.179 (1.065 to 1.306),<0.002[0.061] | 1.272 (1.374 to 1.374),<0.001[0.050] | 1.280 (1.185 to 1.382),<0.001[0.050] | 1.327 (1.265 to 1.390),<0.001[0.032] | |
| **2019** | 1.291 (1.158 to 1.439),<0.001[0.072] | 1.336 (1.452 to 1.452),<0.001[0.057] | 1.284 (1.181 to 1.396),<0.001[0.055] | 1.261 (1.197 to 1.328),<0.001[0.034] | |
| **2020** | 1.182 (1.026 to 1.362),<0.020[0.085] | 1.332 (1.480 to 1.480),<0.001[0.071] | - | - | |
| **2021** | - | - | - | - | |
| **constant** | 0.001 (0.001 to 0.002),<0.001[0.001] | 0.001 (0.001 to 0.001),<0.001[0.001] | 0.002 (0.002 to 0.002),<0.001[0.001] | 0.004 (0.004 to 0.006),<0.001[0.001] | |

a 95% confidence intervals are in brackets; results are reported as incidence rate ratios (IRR) followed by P-values and standard errors in parentheses.

b Coefficients can be interpreted as proportionate changes, for example, patients in the North West had on average 35.4% more A&E visits within 1 to 7 days than .patients in the North East

| Table S20 - Mixed effects logistic regression for patient outcomes (12/12) over time pt3, OR a,b | | | | |
| --- | --- | --- | --- | --- |
|  | **ACSC admissions – same day** | **ACSC admissions – within 1 to 7 days** | **Referrals** | **Tests** |
| **Locum consultations** | 0.996 (0.923 to 1.054),<0.677[0.033] | 0.993 (0.939 to 1.041),<0.890[0.025] | 0.850 (0.842 to 0.858),<0.001[0.004] | 0.805 (0.796 to 0.814),<0.001[0.005] |
| **Gender (reference= male)** | 0.921 (0.888 to 0.8956),<0.001[0.017] | 0.906 (0.881 to 0.931),<0.001[0.013] | 0.927 (0.922 to 0.932),<0.001[0.003] | 0.995 (0.989 to 1.001),<0.120[0.003] |
| **Age** | 1.011 (1.010 to 1.012),<0.001[0.001] | 1.008 (1.007 to 1.009),<0.001[0.001] | 1.008 (1.008 to 1.009),<0.001[0.001] | 0.997 (0.997 to 0.998),<0.001[0.009] |
| **Comorbidity score** | 1.372 (1.352 to 1.393),<0.001[0.010] | 1.376 (1.361 to 1.392),<0.001[0.007] | 0.877 (0.874 to 0.881),<0.001[0.001] | 1.090 (1.086 to 1.094),<0.001[0.002] |
| **Years registered with practice** | 0.998 (0.996 to 0.999),<0.004[0.001] | 0.997 (0.996 to 0.998),<0.001[0.001] | 0.999 (0.998 to 0.999),<0.001[0.001] | 1.000 (0.998 to 1.001),<0.103[0.001] |
| **Patient list size** | 0.999 (0.999 to 1.000),<0.059[0.001] | 0.999 (0.999 to 0.999),<0.006[0.001] | 1.000 (0.999 to 1.001),<0.458[0.002] | 1.000 (1.000 to 1.001),<0.001[0.002] |
| **IMD quintile (1=most deprived)** |  | | | |
| **Quintile 2** | 1.113 (1.047 to 1.183),<0.001[0.035] | 1.103 (1.054 to 1.154),<0.001[0.025] | 0.984 (0.977 to 0.993),<0.001[0.004] | 1.009 (0.999 to 1.019),<0.094[0.005] |
| **Quintile 3** | 1.199 (1.126 to 1.276),<0.001[0.038] | 1.155 (1.102 to 1.210),<0.001[0.027] | 0.963 (0.954 to 0.972),<0.001[0.004] | 1.006 (0.995 to 1.016),<0.300[0.005] |
| **Quintile 4** | 1.295 (1.213 to 1.383),<0.001[0.043] | 1.265 (1.205 to 1.328),<0.001[0.031] | 0.939 (0.929 to 0.948),<0.001[0.005] | 1.017 (1.006 to 1.029),<0.003[0.006] |
| **Quintile 5** | 1.408 (1.313 to 1.510),<0.001[0.050] | 1.289 (1.222 to 1.358),<0.001[0.035] | 0.976 (0.899 to 0.921),<0.001[0.005] | 0.999 (0.986 to 1.012),<0.848[0.006] |
| **Rurality** | 0.968 (0.904 to 1.037),<0.353[0.034] | 0.892 (0.845 to 0.942),<0.001[0.025] | 1.028 (1.014 to 1.040),<0.001[0.007] | 0.977 (0.963 to 0.990),<0.001[0.007] |
| **Region**  **(reference=N. East)** |  | | | |
| **North West** | 0.837 (0.665 to 1.054),<0.130[0.098] | 0.951 (0.773 to 1.168),<0.630[0.100] | 0.942 (0.366 to 2.425),<0.901[0.454] | 1.190 (0.661 to 2.142),<0.562[0.357] |
| **Yorkshire & Humber** | 0.737 (0.547 to 0.993),<0.045[0.112] | 0.887 (0.683 to 1.151),<0.365[0.118] | 0.262 (0.080 to 0.860),<0.027[0.159] | 1.342 (0.653 to 2.758),<0.423[0.493] |
| **Midlands** | 0.925 (0.732 to 1.170),<0.516[0.111] | 1.084 (0.878 to 1.336),<0.454[0.116] | 0.829 (0.317 to 2.169),<0.702[0.407] | 1.187 (0.653 to 2.157),<0.574[0.362] |
| **East of England** | 0.889 (0.697 to 1.135),<0.345[0.111] | 0.968 (0.779 to 1.203),<0.771[0.107] | 0.715 (0.264 to 1.935),<0.509[0.363] | 1.498 (0.809 to 2.772),<0.198[0.470] |
| **London** | 0.812 (0.644 to 1.024),<0.079[0.096] | 0.890 (0.723 to 1.095),<0.269[0.094] | 1.137 (0.443 to 2.919),<0.789[0.547] | 1.733 (0.964 to 3.114),<0.066[0.519] |
| **South East** | 0.792 (0.631 to 0.994),<0.045[0.092] | 0.895 (0.731 to 1.098),<0.288[0.093] | 0.690 (0.274 to 1.739),<0.432[0.325] | 1.078 (0.604 to 1.923),<0.799[0.318] |
| **South West** | 0.703 (0.553 to 0.893),<0.004[0.086] | 0.817 (0.660 to 1.011),<0.063[0.089] | 0.513 (0.195 to 1.353),<0.177[0.254] | 0.903 (0.495 to 1.648),<0.740[0.277] |
| **Year (reference year=2010)** |  | | | |
| **2011** | 1.021 (0.952 to 1.094),<0.564[0.036] | 1.061 (1.008 to 1.116),<0.022[0.027] | 0.925 (0.916 to 0.934),<0.319[0.005] | 0.939 (0.929 to 0.949),<0.001[0.005] |
| **2012** | 1.050 (0.980 to 1.126),<0.166[0.037] | 1.096 (1.042 to 1.153),<0.001[0.028] | 0.869 (0.860 to 0.878),<0.001[0.004] | 0.905 (0.895 to 0.915),<0.001[0.005] |
| **2013** | 1.091 (1.017 to 1.171),<0.015[0.039] | 1.049 (0.995 to 1.106),<0.074[0.028] | 0.882 (0.873 to 0.891),<0.001[0.005] | 0.839 (0.830 to 0.849),<0.001[0.005] |
| **2014** | 1.073 (0.997 to 1.156),<0.062[0.041] | 1.116 (1.057 to 1.178),<0.001[0.031] | 0.914 (0.904 to 0.923),<0.001[0.005] | 0.830 (0.820 to 0.840),<0.001[0.005] |
| **2015** | 1.134 (1.048 to 1.227),<0.002[0.045] | 1.100 (1.037 to 1.167),<0.002[0.033] | 0.944 (0.934 to 0.955),<0.001[0.006] | 0.817 (0.806 to 0.827),<0.001[0.005] |
| **2016** | 1.157 (1.059 to 1.264),<0.001[0.052] | 1.117 (1.044 to 1.194),<0.001[0.038] | 0.977 (0.965 to 0.989),<0.001[0.007] | 0.839 (0.826 to 0.851),<0.001[0.006] |
| **2017** | 1.209 (1.097 to 1.332),<0.001[0.060] | 1.152 (1.070 to 1.239),<0.001[0.043] | 0.971 (0.957 to 0.985),<0.001[0.007] | 0.887 (0.873 to 0.902),<0.001[0.007] |
| **2018** | 1.275 (1.152 to 1.412),<0.001[0.066] | 1.283 (1.190 to 1.384),<0.001[0.050] | 1.039 (1.022 to 1.052),<0.001[0.008] | 0.926 (0.910 to 0.942),<0.001[0.008] |
| **2019** | 1.322 (1.184 to 1.476),<0.001[0.074] | 1.367 (1.261 to 1.483),<0.001[0.057] | 1.015 (0.999 to 1.032),<0.072[0.008] | 0.955 (0.937 to 0.973),<0.001[0.009] |
| **2020** | 1.295 (1.127 to 1.488),<0.001[0.092] | 1.358 (1.225 to 1.505),<0.001[0.071] | 0.656 (0.640 to 0.673),<0.001[0.008] | 0.322 (0.310 to 0.334),<0.001[0.006] |
| **2021** | - | - | 0.757 (0.734 to 0.781),<0.001[0.011] | 0.437 (0.418 to 0.458),<0.001[0.010] |
| **constant** | 0.001 (0.001 to 0.002),<0.001[0.001] | 0.001 (0.001 to 0.001),<0.001[0.001] | 0.026 (0.011 to 0.063),<0.001[0.001] | 0.020 (0.011 to 0.035),<0.001[0.006] |

a 95% confidence intervals are in brackets; results are reported as incidence rate ratios (IRR) followed by P-values and standard errors in parentheses.

b Coefficients can be interpreted as proportionate changes, for example, patients in the North West had on average 19% more tests than .patients in the North East

| Table S21 - Mixed effects logistic regression for safety indicators (5/10) over time pt1, OR a,b | | | | | |
| --- | --- | --- | --- | --- | --- |
|  | **Indicator A** | **Indicator B** | **Indicator C** | **Indicator D** | **Indicator E** |
| **Locum consultations** | **1.116 (1.077 to 1.157),**  **<0.001 [0.020]** | **1.442 (0.939 to 2.217),**  **<0.095 [0.316]** | **1.351 (0.720 to 2.535),**  **<0.349 [0.434]** | **0.772 (0.641 to 0.931),**  **<0.007 [0.074]** | **1.066 (0.790 to 1.438),**  **<0.675 [0.163]** |
| **Gender (reference= male)** | 0.756 (0.736 to 0.777),  <0.001 [0.010] | 0.948 (0.696 to 1.291),  <0.735 [0.149] | 0.821 (0.538 to 1.252),  <0.360 [0.177] | 1.029 (0.902 to 1.175),  <0.665 [0.070] | 1.255 (0.869 to 1.811),  <0.226 [0.235] |
| **Age** | 0.983 (0.981 to 0.985),  <0.001 [0.001] | 0.995 (0.985 to 1.005),  <0.354 [0.005] | 1.019 (1.001 to 1.039),  <0.044 [0.010] | 0.953 (0.948 to 0.959),  <0.001 [0.003] | 1.001 (0.984 to 1.018),  <0.918 [0.008] |
| **Comorbidity score** | 0.992 (0.980 to 1.004),  <0.193 [0.006] | 0.975 (0.843 to 1.127),  <0.731 [0.072] | 0.959 (0.827 to 1.112),  <0.579 [0.073] | 0.874 (0.831 to 0.918),  <0.001 [0.022] | 0.593 (0.496 to 0.710),  <0.001 [0.054] |
| **Years registered with practice** | 0.999 (0.998 to 1.000),  <0.001 [0.001] | 1.001 (0.992 to 1.016),  <0.502 [0.006] | 0.998 (0.984 to 1.011),  <0.721 [0.007] | 1.004 (0.996 to 1.004),  <0.838 [0.002] | 1.013 (1.001 to 1.025),  <0.026 [0.005] |
| **Patient list size** | 1.000 (1.000 to 1.001),  <0.001 [0.001] | 1.000 (1.000 to 1.001),  <0.093 [0.001] | 1.000 (0.999 to 1.000),  <0.475 [0.001] | 1.000 (1.000 to 1.000),  <0.001 [0.001] | 0.999 (0.999 to 1.000),  <0.219 [0.001] |
| **IMD quintile (1=most deprived)** |  | | | | |
| **Quintile 2** | 1.059 (1.020 to 1.100),  <0.003 [0.020] | 1.183 (0.685 to 2.046),  <0.547 [0.331] | 1.923 (0.980 to 3.770),  <0.057 [0.661] | 0.947 (0.780 to 1.149),  <0.582 [0.094] | 1.690 (1.014 to 2.818),  <0.044 [0.441] |
| **Quintile 3** | 1.137 (1.093 to 1.182),  <0.001 [0.023] | 1.130 (0.664 to 1.192),  <0.653 [0.307] | 1.326 (0.695 to 2.533),  <0.392 [0.438] | 0.867 (0.711 to 1.058),  <0.159 [0.088] | 0.990 (0.612 to 1.599),  <0.966 [0.243] |
| **Quintile 4** | 1.171 (1.122 to 1.222),  <0.001 [0.025] | 0.938 (0.557 to 1.580),  <0.810 [0.249] | 1.160 (0.583 to 2.306),  <0.673 [0.407] | 1.055 (0.857 to 1.300),  <0.611 [0.112] | 0.741 (0.400 to 1.371),  <0.339 [0.233] |
| **Quintile 5** | 1.338 (1.275 to 1.404),  <0.001 [0.033] | 1.119 (0.661 to 1.894),  <0.675 [0.301] | 2.007 (0.984 to 4.095),  <0.055 [0.730] | 0.945 (0.753 to 1.186),  <0.627 [0.110] | 0.529 (0.287 to 0.975),  <0.041 [0.165] |
| **Rurality** | 0.787 (0.760 to 0.814),  <0.001 [0.014] | 1.126 (0.699 to 1.814),  <0.625 [0.274] | 1.066 (0.564 to 2.015),  <0.843 [0.346] | 1.261 (1.057 to 1.506),  <0.010 [0.113] | 2.055 (1.253 to 3.370),  <0.004 [0.519] |
| **Region (reference=N. East)** |  | | | | |
| **North West** | 0.842 (0.753 to 0.943),  <0.003 [0.048] | 1.411 (0.584 to 3.407),  <0.444 [0.635] | 1.166 (0.277 to 4.912),  <0.834 [0.856] | 3.666 (1.773 to 7.580),  <0.001 [1.358] | 0.164 (0.047 to 0.566),  <0.004 [0.104] |
| **Yorkshire & Humber** | 2.057 (1.793 to 2.361),  <0.001 [0.144] | 1.483 (0.432 to 5.091),  <0.531 [0.933] | 4.520 (0.691 to 29.585),  <0.116 [4.333] | 3.697 (1.600 to 8.544),  <0.002 [1.580] | 2.975 (0.606 to 14.607),  <0.179 [2.416] |
| **Midlands** | 0.828 (0.739 to 0.928),  <0.001 [0.048] | 1.773 (0.705 to 4.461),  <0.224 [0.835] | 1.301 (0.302 to 5.604,  <0.724 [0.969] | 3.020 (1.452 to 6.281),  <0.003 [1.128] | 1.142 (0.313 to 4.168),  <0.841 [0.754] |
| **East of England** | 1.067 (0.948 to 1.200),  <0.281 [0.064] | 2.441 (0.880 to 6.769),  <0.086 [1.270] | 2.317 (0.500 to 10.724),  <0.282 [1.811] | 2.424 (1.149 to 5.116),  <0.020 [0.924] | 1.755 (0.470 to 6.552),  <0.403 [1.180] |
| **London** | 0.990 (0.882 to 1.111),  <0.866 [0.058] | 0.904 (0.359 to 2.276),  <0.830 [0.426] | 1.507 (0.347 to 6.538),  <0.584 [1.128] | 2.472 (1.184 to 5.162),  <0.016 [0.929] | 0.214 (0.058 to 0.789),  <0.021 [0.142] |
| **South East** | 0.912 (0.816 to 1.019),  <0.104 [0.052] | 1.946 (0.801 to 4.727),  <0.141 [0.881] | 1.607 (0.377 to 6.849),  <0.522 [1.188] | 3.388 (1.644 to 6.982),  <0.001 [1.250] | 0.670 (0.190 to 2.360),  <0.532 [0.430] |
| **South West** | 0.939 (0.837 to 1.052),  <0.277 [0.055] | 1.881 (0.739 to 4.786),  <0.185 [0.896] | 1.706 (0.375 to 7.766),  <0.490 [1.319] | 4.018 (1.933 to 8.353),  <0.001 [1.500] | 1.684 (0.466 to 6.085),  <0.426 [1.104] |
| **Year (reference year=2010)** |  | | | | |
| **2011** | 0.800 (0.776 to 0.826),  <0.001 [0.012] | 0.846 (0.562 to 1.272),  <0.422 [0.176] | 0.991 (0.609 to 1.614),  <0.973 [0.246] | 1.120 (0.955 to 1.315),  <0.164 [0.091] | 0.506 (0.374 to 0.683),  <0.001 [0.078] |
| **2012** | 0.676 (0.654 to 0.698),  <0.001 [0.011] | 1.166 (0.749 to 1.815),  <0.496 [0.263] | 1.732 (0.996 to 3.013),  <0.052 [0.489] | 1.005 (0.852 to 1.186),  <0.953 [0.085] | 0.464 (0.336 to 0.639),  <0.001 [0.076] |
| **2013** | 0.609 (0.589 to 0.630),  <0.001 [0.010] | 1.013 (0.627 to 1.637),  <0.959 [0.248] | 2.071 (1.157 to 3.707),  <0.014 [0.615] | 0.886 (0.746 to 1.051),  <0.165 [0.077] | 0.491 (0.359 to 0.671),  <0.001 [0.078] |
| **2014** | 0.577 (0.556 to 0.598),  <0.001 [0.011] | 1.134 (0.668 to 1.925),  <0.642 [0.306] | 1.802 (0.943 to 3.442),  <0.075 [0.595] | 0.960 (0.805 to 1.145),  <0.651 [0.086] | 0.523 (0.373 to 0.733),  <0.001 [0.090] |
| **2015** | 0.484 (0.464 to 0.504),  <0.001 [0.010] | 0.882 (0.493 to 1.578),  <0.673 [0.262] | 2.867 (1.371 to 5.992),  <0.005 [1.078] | 0.857 (0.711 to 1.032,  <0.104 [0.082] | 0.468 (0.322 to 0.680),  <0.001 [0.089] |
| **2016** | 0.422 (0.402 to 0.442),  <0.001 [0.011] | 1.181 (0.578 to 2.416),  <0.648 [0.431] | 3.066 (1.298 to 6.993),  <0.011 [1.295] | 0.793 (0.646 to 0.973),  <0.026 [0.083] | 0.172 (0.109 to 0.271),  <0.001 [0.040] |
| **2017** | 0.391 (0.370 to 0.414),  <0.001 [0.011] | 0.835 (0.352 to 1.987),  <0.683 [0.369] | 1.009 (0.363 to 2.805),  <0.986 [0.526] | 0.623 (0.496 to 0.782),  <0.001 [0.072] | 0.139 (0.087 to 0.220),  <0.001 [0.033] |
| **2018** | 0.351 (0.330 to 0.373),  <0.001 [0.011] | 0.940 (0.332 to 2.661),  <0.907 [0.499] | 1.704 (0.473 to 6.132),  <0.415 [1.113] | 0.383 (0.294 to 0.501),  <0.001 [0.052] | 0.063 (0.036 to 0.111),  <0.001 [0.018] |
| **2019** | 0.289 (0.269 to 0.311),  <0.001 [0.011] | 1.021 (0.330 to 3.162),  <0.971 [0.589] | 0.266 (0.051 to 1.374),  <0.114 [0.223] | 0.319 (0.234 to 0.433),  <0.001 [0.050] | 0.120 (0.065 to 0.221),  <0.001 [0.037] |
| **2020** | 0.316 (0.287 to 0.349),  <0.001 [0.016] | 1.713 (0.418 to 7.028),  <0.455 [1.234] | 9.810 (0.772 to 124.623),  <0.078 [12.722] | 0.223 (0.143 to 0.347),  <0.001 [0.050] | 0.020 (0.009 to 0.045),  <0.001 [0.008] |
| **2021** | 0.382 (0.331 to 0.441),  <0.001 [0.028] | 22.005 (0.923 to 524.69),  <0.056 [35.607] | - | 0.285 (0.148 to 0.545),  <0.001 [0.094] | 0.010 (0.001 to 0.068),  <0.001 [0.010] |
| **constant** | 13.369 (11.145 to 16.036),  <0.001 [0.005] | 1.402 (0.436 to 4.515),  <0.571 [0.836] | 0.011 (0.013 to 0.080),  <0.001 [0.010] | 0.003 (0.001 to 0.007),  <0.001 [0.013] | 180.80 (29.810 to 1096.55),  <0.001 [166.27] |

a 95% confidence intervals are in brackets; results are reported as incidence rate ratios (IRR) followed by P-values and standard errors in parentheses.

b Coefficients can be interpreted as proportionate changes, for example, patients in the North West had on average 15.8% fewer consultations where a prescribing error for indicator A was triggered than patients in the North East

| Table S22 - Mixed effects logistic regression for safety indicators (10/10) over time pt2, OR a,b | | | | | |
| --- | --- | --- | --- | --- | --- |
|  | **Indicator F** | **Indicator G** | **Indicator H** | **Indicator I** | **Indicator J** |
| **Locum consultations** | **0.992 (0.915 to 1.076),**  **<0.852 [0.041]** | **0.994 (0.906 to 1.090),**  **<0.894 [0.047]** | **0.888 (0.848 to 0.929),**  **<0.001 [0.021]** | **0.958 (0.812 to 1.130),**  **<0.613 [0.010]** | **0.487 (0.193 to 1.229),**  **<0.128 [0.230]** |
| **Gender (reference= male)** | 0.668 (0.555 to 0.806),  <0.001 [0.063] | 1.565 (1.416 to 1.728),  <0.001 [0.080] | 1.070 (1.007 to 1.136),  <0.001 [0.032] | 0.962 (0.850 to 1.087),  <0.534 [0.007] | 0.568 (0.279 to 1.156),  <0.119 [0.206] |
| **Age** | 0.965 (0.958 to 0.972),  <0.001 [0.004] | 0.933 (0.930 to 0.936),  <0.001 [0.002] | 1.008 (1.006 to 1.009),  <0.001 [0.001] | 0.966 (0.961 to 0.970),  <0.001 [0.001] | 1.097 (1.034 to 1.164),  <0.002 [0.033] |
| **Comorbidity score** | 0.556 (0.506 to 0.611),  <0.001 [0.026] | 0.665 (0.637 to 0.693),  <0.001 [0.014] | 1.106 (1.077 to 1.134),  <0.001 [0.014] | 0.891 (0.854 to 0.930),  <0.001 [0.003] | 0.889 (0.698 to 1.130),  <0.337 [0.109] |
| **Years registered with practice** | 1.008 (1.002 to 1.014),  <0.006 [0.003] | 0.997 (0.994 to 1.002),  <0.151 [0.001] | 1.005 (0.998 to 1.003),  <0.655 [0.001] | 1.001 (0.998 to 1.004),  <0.509 [0.001] | 1.021 (0.999 to 1.042),  <0.052 [0.011] |
| **Patient list size** | 1.000 (1.000 to 1.001),  <0.002 [0.001] | 1.000 (1.000 to 1.001),  <0.818 [0.001] | 0.999 (0.999 to 1.000),  <0.001 [0.001] | 0.999 (0.999 to 1.000),  <0.656 [0.001] | 1.000 (0.998 to 1.001),  <0.667 [0.001] |
| **IMD quintile (1=most deprived)** |  | | | | |
| **Quintile 2** | 0.964 (0.738 to 1.260),  <0.791 [0.132] | 1.035 (0.893 to 1.200),  <0.647 [0.078] | 0.861 (0.788 to 0.941),  <0.001 [0.039] | 1.093 (0.906 to 1.318),  <0.353 [0.009] | 0.806 (0.296 to 2.190),  <0.672 [0.411] |
| **Quintile 3** | 0.836 (0.638 to 1.095),  <0.195 [0.115] | 0.932 (0.803 to 1.083),  <0.351 [0.071] | 0.748 (0.684 to 0.819),  <0.001 [0.034] | 0.962 (0.794 to 1.166),  <0.695 [0.010] | 0.441 (0.174 to 1.120),  <0.085 [0.210] |
| **Quintile 4** | 0.798 (0.599 to 1.064),  <0.124 [0.117] | 0.934 (0.804 to 1.085),  <0.375 [0.071] | 0.736 (0.675 to 0.804),  <0.001 [0.033] | 1.057 (0.867 to 1.288),  <0.582 [0.012] | 0.816 (0.304 to 2.190),  <0.686 [0.411] |
| **Quintile 5** | 0.400 (0.285 to 0.560),  <0.001 [0.069] | 0.892 (0.763 to 1.043),  <0.151 [0.071] | 0.763 (0.695 to 0.837),  <0.001 [0.036] | 1.178 (0.958 to 1.448),  <0.120 [0.015] | 1.724 (0.517 to 5.751),  <0.375 [1.059] |
| **Rurality** | 1.264 (1.005 to 1.591,  <0.045 [0.148] | 0.975 (0.846 to 1.122),  <0.721 [0.070] | 0.824 (0.752 to 0.902),  <0.001 [0.038] | 0.959 (0.809 to 1.137),  <0.631 [0.012] | 1.288 (0.481 to 3.448),  <0.615 [0.647] |
| **Region (reference=N. East)** |  | | | | |
| **North West** | 0.149 (0.086 to 0.259),  <0.001 [0.042] | 1.085 (0.761 to 1.548),  <0.651 [0.196] | 0.392 (0.325 to 0.472),  <0.001 [0.037] | 2.269 (1.277 to 4.031),  <0.165 [0.168] | 0.042 (0.007 to 2.375),  <0.124 [0.086] |
| **Yorkshire & Humber** | 0.753 (0.397 to 1.428),  <0.385 [0.246] | 1.027 (0.626 to 1.684),  <0.916 [0.259] | 0.877 (0.668 to 1.151),  <0.001 [0.122] | 2.231 (1.126 to 4.423),  <0.021 [0.177] | 0.023 (0.002 to 1.892),  <0.094 [0.052] |
| **Midlands** | 0.263 (0.151 to 0.457),  <0.001 [0.074] | 0.894 (0.620 to 1.289),  <0.547 [0.167] | 0.410 (0.339 to 0.496),  <0.001 [0.040] | 3.084 (1.725 to 5.514),  <0.001 [0.190] | 0.029 (0.001 to 1.677),  <0.087 [0.059] |
| **East of England** | 0.229 (0.128 to 0.411),  <0.001 [0.068] | 0.787 (0.537 to 1.155),  <0.222 [0.154] | 0.497 (0.406 to 0.609),  <0.001 [0.052] | 1.824 (0.991 to 3.357),  <0.053 [0.230] | 0.111 (0.015 to 7.694),  <0.309 [0.240] |
| **London** | 0.055 (0.029 to 0.103),  <0.001 [0.018] | 0.899 (0.623 to 1.297),  <0.569 [0.168] | 0.827 (0.684 to 0.999),  <0.049 [0.080] | 2.273 (1.262 to 4.094),  <0.006 [0.171] | 0.019 (0.001 to 1.115),  <0.056 [0.039] |
| **South East** | 0.209 (0.123 to 0.357),  <0.001 [0.057] | 0.861 (0.603 to 1.228),  <0.409 [0.156] | 0.347 (0.288 to 0.418),  <0.059 [0.033] | 2.466 (1.388 to 4.382),  <0.002 [0.199] | 0.050 (0.008 to 2.898),  <0.148 [0.103] |
| **South West** | 0.681 (0.399 to 1.164),  <0.161 [0.186] | 0.907 (0.622 to 1.321),  <0.609 [0.174] | 0.368 (0.302 to 0.448),  <0.145 [0.037] | 2.737 (1.526 to 4.910),  <0.001 [0.218] | 0.026 (0.001 to 1.550),  <0.080 [0.054] |
| **Year (reference year=2010)** |  | | | | |
| **2011** | 0.815 (0.748 to 0.888),  <0.001 [0.036] | 0.876 (0.778 to 0.986),  <0.028 [0.053] | 0.760 (0.732 to 0.790),  <0.001 [0.015] | 0.902 (0.779 to 1.043),  <0.163 [0.010] | 0.826 (0.340 to 2.006),  <0.673 [0.374] |
| **2012** | 0.687 (0.627 to 0.753),  <0.001 [0.032] | 0.805 (0.713 to 0.908),  <0.001 [0.050] | 0.648 (0.623 to 0.674),  <0.001 [0.013] | 0.653 (0.554 to 0.770),  <0.001 [0.009] | 0.911 (0.366 to 2.269),  <0.842 [0.424] |
| **2013** | 0.595 (0.541 to 0.654),  <0.001 [0.029] | 0.785 (0.696 to 0.887),  <0.001 [0.049] | 0.478 (0.459 to 0.498),  <0.001 [0.010] | 0.669 (0.565 to 0.792),  <0.001 [0.010] | 1.423 (0.528 to 3.835),  <0.485 [0.720] |
| **2014** | 0.594 (0.537 to 0.658),  <0.001 [0.031] | 0.753 (0.664 to 0.853),  <0.001 [0.048] | 0.387 (0.370 to 0.404),  <0.001 [0.009] | 0.686 (0.575 to 0.820),  <0.001 [0.010] | 0.857 (0.320 to 2.292),  <0.758 [0.430] |
| **2015** | 0.474 (0.424 to 0.529),  <0.001 [0.027] | 0.758 (0.664 to 0.865),  <0.001 [0.051] | 0.274 (0.260 to 0.288),  <0.001 [0.007] | 0.510 (0.416 to 0.625,  <0.001 [0.011] | 1.361 (0.422 to 4.394),  <0.606 [0.814] |
| **2016** | 0.359 (0.318 to 0.406),  <0.001 [0.022] | 0.726 (0.630 to 0.837),  <0.001 [0.053] | 0.228 (0.214 to 0.241),  <0.001 [0.007] | 0.499 (0.399 to 0.624),  <0.001 [0.012] | 2.422 (0.615 to 9.541),  <0.206 [1.694] |
| **2017** | 0.326 (0.286 to 0.373),  <0.001 [0.022] | 0.703 (0.604 to 0.819),  <0.001 [0.055] | 0.171 (0.160 to 0.184),  <0.001 [0.006] | 0.402 (0.309 to 0.523),  <0.001 [0.013] | 3.228 (0.522 to 19.954),  <0.207 [3.000] |
| **2018** | 0.332 (0.287 to 0.384),  <0.001 [0.025] | 0.721 (0.612 to 0.849),  <0.001 [0.060] | 0.121 (0.111 to 0.132),  <0.001 [0.005] | 0.233 (0.168 to 0.324),  <0.001 [0.013] | 2.495 (0.329 to 18.935),  <0.377 [2.580] |
| **2019** | 0.265 (0.225 to 0.312),  <0.001 [0.022] | 0.784 (0.658 to 0.934),  <0.006 [0.070] | 0.085 (0.077 to 0.095),  <0.001 [0.005] | 0.323 (0.234 to 0.446),  <0.001 [0.013] | 0.978 (0.179 to 5.353),  <0.979[0.848] |
| **2020** | 0.346 (0.281 to 0.427),  <0.001 [0.037] | 0.616 (0.486 to 0.781),  <0.001 [0.075] | 0.049 (0.041 to 0.057),  <0.001 [0.004] | 0.262 (0.171 to 0.403),  <0.001 [0.017] | 4.105 (0.273 to 61.700),  <0.307 [5.676] |
| **2021** | 0.497 (0.380 to 0.650),  <0.001 [0.068] | 0.539 (0.373 to 0.779),  <0.001 [0.101] | 0.023 (0.017 to 0.031),  <0.001 [0.003] | 0.378 (0.214 to 0.668),  <0.001 [0.023] | 2.247 (0.040 to 126.372),  <0.694 [4.619] |
| **constant** | 2,624.92(1,285.25 to 5,361.01),  <0.001 [956.37] | 3.632 (2.393 to 5.514),  <0.001 [0.773] | 0.001 (0.001 to 0.001),  <0.001 [0.001] | 0.010 (0.005 to 0.020),  <0.001 [0.004] | 48.753 (0.111 to 21,361.8),  <0.210 [151.30] |

a 95% confidence intervals are in brackets; results are reported as incidence rate ratios (IRR) followed by P-values and standard errors in parentheses.

b Coefficients can be interpreted as proportionate changes, for example, patients in the North West had on average 85.1% fewer consultations where a prescribing error for indicator F was triggered than patients in the North East

| **Effects of locum consultations on patient outcomes** | |
| --- | --- |
| Practice revisits | 0.87 (0.87 to 0.88), <0.001 [0.003] |
| Antibiotic prescriptions | 1.21 (1.21 to 1.22), <0.001 [0.004] |
| Strong opioid prescriptions | 1.08 (1.07 to 1.09), <0.001 [0.007] |
| Hypnotic prescriptions | 0.97 (0.94 to 0.99), <0.002 [0.010] |
| Emergency admissions, same day | 0.95 (0.88 to 1.02), <0.126 [0.034] |
| Emergency admissions within 1 to 7 days | 1.00 (0.94 to 1.05), <0.861 [0.027] |
| A&E visits, same day | 1.02 (0.97 to 1.07), <0.408 [0.026] |
| A&E visits, within 1 to 7 days | 1.05 (1.02 to 1.08), <0.001 [0.015] |
| ACSC admissions, same day | 0.99 (0.92 to 1.06), <0.793 [0.036] |
| ACSC admissions, within 1 to 7 days | 0.99 (0.95 to 1.05), <0.839 [0.026] |
| Referrals | 0.85 (0.85 to 0.86),< 0.001 [0.004] |
| Tests | 0.80 (0.80 to 0.81),< 0.001 [0.005] |
| **Effects of locum consultations on potentially hazardous prescribing indicators** | |
| Indicator A | 1.11 (1.07 to 1.16), < 0.001 [0.020] |
| Indicator B | 1.47 (0.96 to 2.27), < 0.079 [0.325] |
| Indicator C | 1.38 (0.74 to 2.60), < 0.312 [0.444] |
| Indicator D | 0.77 (0.64 to 0.94), < 0.008 [0.075] |
| Indicator E | 1.09 (0.80 to 1.47), < 0.587 [0.167] |
| Indicator F | 0.99 (0.92 to 1.09), < 0.992 [0.042] |
| Indicator G | 0.97 (0.89 to 1.07), < 0.601 [0.047] |
| Indicator H | 0.88 (0.84 to 0.93), < 0.001 [0.021] |
| Indicator I | 0.97 (0.83 to 1.14), < 0.709 [0.078] |
| Indicator J | 0.65 (0.41 to 1.02), < 0.062 [0.150] |

## Table S23 Mixed effects logistic regression for patient outcomes and potentially hazardous prescribing indicators over time (excluding 2020-2022), odds ratios
